# Does an aerobic endurance programme have an influence on information processing in migraineurs?

**DOI:** 10.1186/1129-2377-15-11

**Published:** 2014-02-14

**Authors:** Claudia H Overath, Stephanie Darabaneanu, Marie C Evers, Wolf-Dieter Gerber, Melanie Graf, Armin Keller, Uwe Niederberger, Henrik Schäl, Michael Siniatchkin, Burkhard Weisser

**Affiliations:** 1 Institute of Medical Psychology, University Clinic of Schleswig-Holstein, Campus Kiel, Diesterwegstraße 10 – 12, 24113 Kiel, Germany; 2 Institute of Sports Medicine, Christian-Albrechts-University, Kiel, Germany; 3 Department of Medicine, Institute of Medical Psychology and Medical Sociology, University of Rostock, Rostock, Germany

**Keywords:** Migraine, Sport, Exercise, Jogging, Walking, Contingent negative variation, CNV, Habituation, Dishabituation

## Abstract

**Background:**

Migraine is a disorder of central information processing which is characterized by a reduced habituation of event-related potentials. There might be positive effects of aerobic exercise on brain function and pain. The aim of this study was to investigate the influence of exercise on information processing and clinical course of migraine.

**Methods:**

33 patients completed a ten-week aerobic exercise programme. To examine the influence of the treatment on information processing and attention, Trail Making Test (TMT) A and B, d2-Letter Cancellation Test (LCT) and recordings of the Contingent Negative Variation (CNV) were performed before and after the training.

**Results:**

Patients showed a significant reduction of the migraine attack frequency, the iCNV-amplitude and the processing time for TMT-A and TMT-B after treatment. Moreover, there was a significant increase of the habituation and positive changes in parameters of attention (d2-LCT) after the training.

**Conclusions:**

This study demonstrates that aerobic exercise programme influences central information processing and leads to clinical effects on the migraine symptomatology. The results can be interpreted in terms of an improvement of a dysfunctional information processing and a stimulus selection under aerobic exercise.

## Background

Migraine is a disorder of central information processing. On the behavioural level, patients with migraine demonstrate an increased attention performance which may be illustrated by, for example, smaller hit or interictal saccadic reaction times [1,2]. On the neurophysiological level, migraineurs are characterized by a reduced habituation of evoked and event-related potentials [3,4]. The contingent negative variation (CNV) is an event-related slow cortical potential which occurs between two stimuli while the subjects is preparing for a response. The CNV represents both attentional performance and information processing during repetitive stimulation and can be segmented in three components: the early component (initial or iCNV, 550-750 ms after S1), the late component (late or lCNV, 2800-3000 ms after S1) and the total component (total or tCNV, 0-3000 ms after S1). There are a number of studies which have revealed increased amplitudes and reduced habituation of the CNV, especially of its early component, in patients suffering from migraine [3,5-8]. It seems likely that the amplitude and habituation of the iCNV are closely related to pathogenetic mechanisms of migraine attacks. The most pronounced increase of the iCNV amplitude and the most pronounced loss of the iCNV habituation were observed immediately before a migraine attack [9]. Therefore, the CNV amplitude seems to be a surrogate marker for the chance to develop a migraine attack. In line with this observations, anti-migraine medication such as ß-blockers, anti-epileptic drugs and calcium antagonists [10-12] as well as psychotherapy [13] have caused both an improvement of the clinical course of migraine and a normalization of the iCNV amplitude and habituation in migraineurs. Based on these studies, it can be suggested that effective treatments of migraine should cause clinical improvement through normalization of CNV parameters.

Several studies have demonstrated a good clinical efficacy of sports in migraine [14-16]. Positive effects of aerobic exercise on the number and the intensity of migraine attacks have been repeatedly shown [17-28]. An increase of the fitness level is one possible predictor for the reduction of migraine frequency [17]. It is unclear, however, which mechanisms may explain the clinical efficacy of the aerobic exercise. More recent studies provided an evidence for a significant correlation between fitness and attention [29,30], indicating that attentional properties can be influenced by aerobic fitness training in healthy subjects [31,32]. Additionally, fitter subjects were characterized by lower CNV amplitudes [33]. Thus, the normalization of attentional performance and of event-related potentials may be involved in the clinical effect of sports in migraine [29,30,34]. It could be hypothesized that aerobic exercise would reduce the number of migraine attacks, normalize the amplitude and habituation of the iCNV and improve attentional performance. This hypothesis will be tested in the present open label study.

### Methods and subjects

Migraine patients completed a ten- week aerobic endurance programme. Eight weeks before the treatment (phase 1), during the intervention and eight weeks after the training (phase 3), the participants completed migraine-diaries which recorded the frequency, intensity and duration of migraine attacks. After the first training period and before the third test (phase 3), information and attention processing were measured using paper pencil tests (TMT-A, TMT-B and d2-LCT) and cortical information processing was assessed using the CNV.

### Subjects

52 patients suffering from migraine were recruited by newspaper advertisements and in neurological praxes. The following inclusion criteria were considered: 1) adult patients (>18 years old) of both sexes; 2) migraine with or without aura; 3) at least two migraine attacks per month and duration of migraine of at least five days per month; 4) no endurance training before the study; 5) no risk that exercise would provoke a migraine attack. Structured headache interviews were performed with all participants and migraine diagnosis was made by an experienced neurologist according to the revised criteria of the International Headache Society IHS [35], code 1.2.0 and 1.2.1. Additionally, a prospective daily headache diary was used to assess headache characteristics over an eight-week-period before the patients were included in the study. The diagnosis of migraine with and without aura was validated by the headache diary in all patients.

28 participants (63.5%) completed the training and the measurements. Clinical and demographic characteristics of these patients are given Table 1. Neurological and routine medical examinations revealed no health problems (including acute infection) other than primary headaches in all participants. None of the subjects presented with any psychiatric disorder, which would have fulfilled the diagnostic criteria of DSM-IV-TR [36]. None of the participants used any medication or took part in any non-pharmacological treatment programs for at least six months prior to the investigation. None of the patients took acute anti-migraine medication more than 2 times a months. None of the subjects suffered from chronic tension-type headache (episodic tension-type headaches were permitted but not controlled in the headache diaries). Experiments were conducted during the headache-free interval. None of the subjects had a hearing impairment or had drunk alcohol during the 3 days before investigation.

**Table 1:** Clinical characteristics of the patient group

**N**	**Male (%)**	**Female (%)**	**MO (%)**	**MA (%)**	**Age (SD)**	**Duration of disease (SD)**
28	5 (17.9)	23 (82.1)	22 (87.6)	6 (21.4)	43.4 (9.7)	19.9 (9.9)

MO = migraine without aura; MA = migraine with aura.

The study was permitted by the Ethic Committee of the Faculty of Medicine, University of Kiel, Germany. All participants were instructed about the study and written informed consent according to the Declaration of Helsinki (current version, 1996) on biomedical research involving human subjects (Tokyo amendment) was obtained.

### Description of the training

Before and after the end of the training period, physical working capacity was measured by participants walking or jogging until a heart rate of 150 beats per minute (BPM) was reached. Than it was compared how fast in kilometers per hours (km/h) the person moved (physical working capacity (PWC 150). The participants completed a supervised ten-week aerobic endurance programme (walking or interval jogging) three times a week. The training was standardised as follows: warming up (five to ten minutes), 30 minutes walking or jogging and cool down incl. stretching (five to ten minutes). The jogging program was performed according to an interval endurance method with a steady increase of running-time compared to walking-time. After five weeks the participants just kept on running without walking intervals in-between. The training was heart rate oriented, so that it was realized in the aerobic area. This means that each person had been communicated a pulse rate area which the participants shouldn’t surpass.

### CNV measurement

The CNV was measured by a two-stimulus-paradigm [37]. The stimuli were presented by “E-Prime” [38], E-Studio; Psychology Software Tools, Inc., Pittsburgh, USA. The participants were instructed to lie calmly in a comfortable chair and keep the eyes open without blinking. A CNV session consisted of 32 trials in which the subject was to react immediately to the imperative stimulus (GO-response). In addition, eight trials were randomly presented in which no reaction was expected (NO-GO-response). The warning stimulus (S1) for the GO-response had a frequency of *f* = 1000 Hz and lasted 100 ms. The warning tone for the NO-GO-response had a frequency of *f* = 200 Hz. The imperative stimulus (S2) had a frequency of *f* = 2500 Hz, lasted a maximum of 3000 ms and was deactivated by pressing the button. Reaction time was defined as the period between the onset of S2 and the pressing of the button. S1 and S2 pairs were offered at random intervals of 10–15 s. The interstimulus interval (ISI) was 3 s.

The EEG was continuously recorded from Cz 10 – 20 system, [39] with a reference located at the left mastoid and grounding placed behind the right ear. Sintered Ag/AgCl ring electrodes were attached using the “BrainCap” (Falk-Minow Services, Herrsching-Breitbrunn, Germany), which is part of the EEG recording system “BrainAmp-MR” (Brainproducts Co., Munich, Germany). Vertical electro-oculogram (VEOG) was recorded from electrodes placed 1 cm above and below the left eye. Electrode impedance was kept below 10 kOhm. The EEG data were obtained using BrainVision recorder software version 1.0 [40]. Data were transmitted from the high-input impedance amplifier (250 Hz low-pass filter, 10 s time constant, 16-bit resolution, dynamic range 16 · 38 mV, sampling rate 250 Hz).

### Data analysis

Raw data were analyzed using BrainVision Analyzer software version 1.0 [Brain Products Co., Munich, 40]. The recordings were transformed to an average reference, corrected automatically for eye movements and blinks by the algorithm of Gratton and Coles (Brain Vision Analyzer). The movement-related artefacts were rejected semi-automatically if the signal amplitude exceeded 100 μV. This step was controlled by visual inspection, and remaining artefacts were removed manually. EEG signals were segmented into units of 5000 ms (1000 ms before S1 to 1000 ms after S2) and filtered by a high-pass-filter (30 Hz, 13 dB/oct) and a notch-filter (50 Hz). Baseline correction was made for intervals 1000 ms before S1.

The GO-trials were averaged and the amplitudes of the total CNV, the iCNV and lCNV components were calculated. The total CNV was assessed between 500 ms and 3000 ms following S1. The iCNV was defined as the mean amplitude in a window of 200 ms around the maximal amplitude of the expectancy wave between 550–750 ms after S1 (14). The lCNV was the mean amplitude during the 200 ms preceding S2. The iCNV is with a retest-reliability of 0,855 the most reliable value of this three CNV components (tCNV: 0.68, lCNV: 0.63) [41]. Each recording was divided into eight blocks of four consecutive trials to determine the course of habituation and trends in the early CNV amplitudes. Habituation was indicated by a negative, whereas dishabituation was marked by a positive slope as calculated by linear regression (*y = ax + b*, where *a* is the slope of habituation and *b* the intercept of linear regression) [9].

### Measurement of executive functions

For measurement of cognitive flexibility, the Trail Making Test (TMT), form A and form B, was used [42]. In form A information processing velocity is measured. Participants had the task to connect numbers from 1 to 25, which are randomly printed on a sheet of paper. In form B, which calculated a mass of split attention, numbers from 1 to 13 and letter from A to L had to be connected. The connection had to be done in ascending order (1-2-3 etc. or 1-A-2-B- 3-C etc.) and as quickly as possible. Measured dependent variables were the processing time and a calculated percentile rank (PR).

The d2-letter cancellation test (internal consistency 0.93 - 0.98; stability 0.89 - 0.94) were used for measurements for individual attention and concentration ability. In particular the ability to allocate relevant intern and extern stimuli selectively was measured [43]. The challenge of the test is based upon time pressure and the monotone repetition of the tasks. The test includes 14 lines with “d” and “p”. This d’s and p’s are marked with different numbers of strokes. Participants were requested to cancel successive each d with two strokes in each line as quickly as possible and with the fewest number of mistakes (from left to right). The participant has to start with the first line and after 20 seconds the examiner told the participant to switch to the next line. In this way the working speed (throughput) and the accuracy (avoidance of mistakes) was measured. For analysis values as total number of finished items, total number of mistakes, total number of finished items less the number of mistakes and a so called concentration-efficiency value were calculated.

### Statistical analysis

All data was tested for normal distribution using Kolmogorov-Smirnov-test and for homogeneity of variance using Levene-test. As the data were normally distributed, t-tests for depended samples were chosen for the calculation of differences before and after training. Product–moment Pearson’s correlations between neurophysiological, clinical and neuropsychological measures were calculated. Statistical analyses were performed using SPSS 18 (SPSS Inc., Chicago, USA) with a significant level of 5% for all tests. Bonferroni adjustment was applied to correct for multiple comparisons. In this manuscript only results concerning the iCNV will be reported. This limitation of data presentation is supported by the literature which has shown that only the changes of the iCNV are relevant for the pathogenesis of migraine [3,5-12]. Indeed, also in this study we did not find any significant effect of treatment for the tCNV and lCNV.

## Results

### Clinical effects of exercise

After the training, there was a significant reduction of both the numberx of days with migraine (t(27) = 2.35; p = .01) and the number of migraine attacks per month (t(27) = 3.04; p = .001) compared with the clinical course of migraine before the training. On average, patients were suffering from migraine for 6.16 ± 2,49 days/month and 3.98 ±1 ,94 attacks/month before aerobic training. After the training, patients suffered from migraine on average 5.27 ± 3,25 days/month and presented with 3.16 ± 1,63 attacks/month. In summary, the patients demonstrated a reduction of days with migraine of approximately 14.5 percent and a reduction of migraine attacks/month of approximately 20.6 percent.

### Executive functions

**Figure 1:**
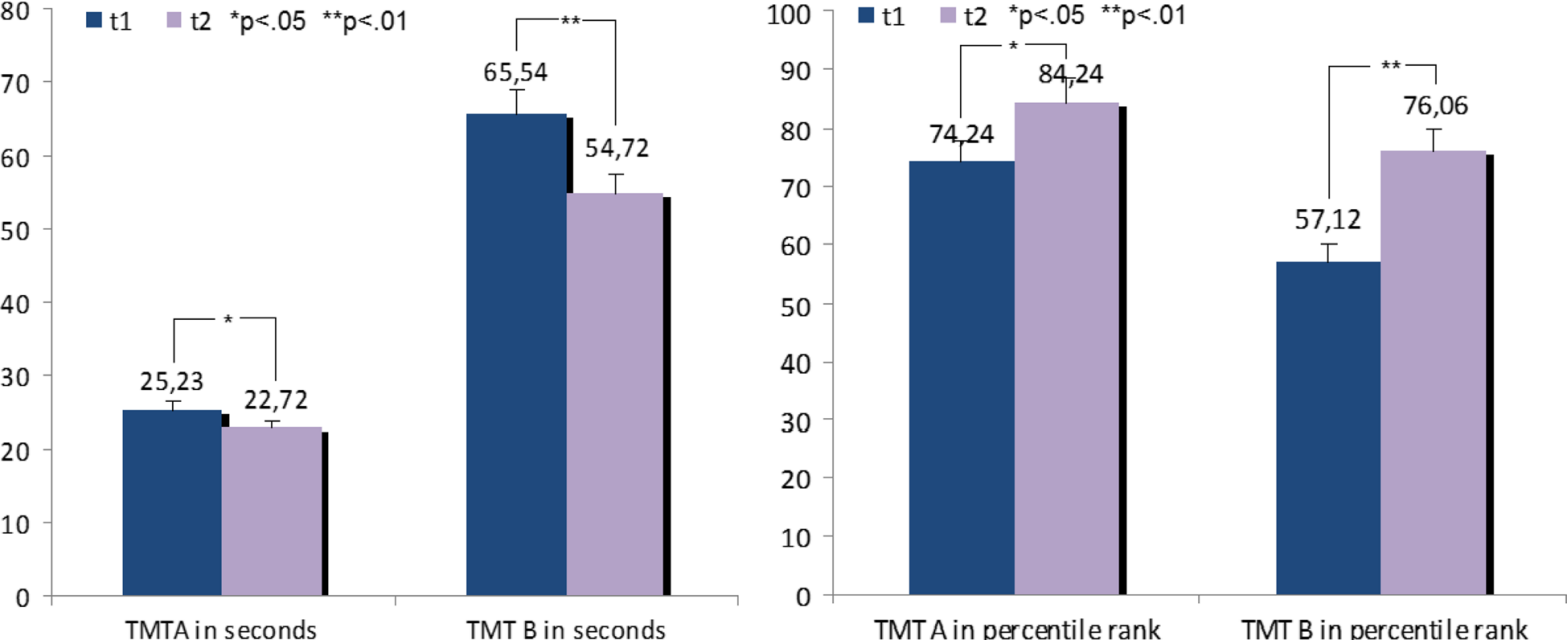
**Differences of means for TMT-A and TMT-B before the training (t1) and after the training (t2).** On the left demonstrated in seconds and on the right in percentile rank.

**Figure 2:**
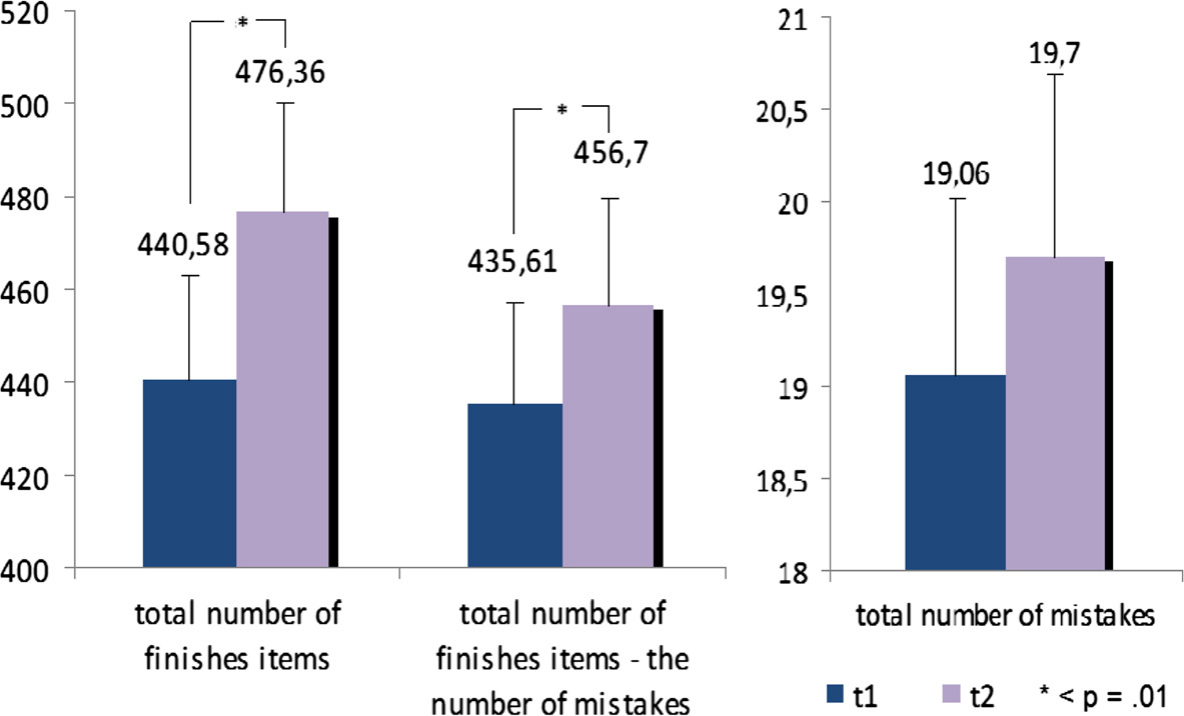
Differences of means for parameters of d2-LCT before the training (t1) and after the training (t2).

Table 2 and Figure 1 demonstrate significant effects of processing time and in percentile rank for TMT-A and TMT-B. Processing time of TMT-A t(32) = 1.79; p = .04) and TMT-B (t(32) = 3.30; p = .001) reduced and percentile ranks of TMT-A (t(32) = −2.10; p = .02) and TMT-B (t(32) = −4.08; p = .001) increased significantly after the training. Note that the percentile rank of TMT-A before the training was 74.2, nearly above-average. After the training, the number of finished items of the d2-letter cancellation test was increased (t(32) = −2.68; p = .01). The “total number of finished items less the number of mistakes” (t(32) = −2.80; p = .01) showed an increase at the second measurement whereas the “total number of mistakes” didn’t changed significantly (t(32) = −.35; p = .37). Table 2 and Figure 2 demonstrate these results.

**Table 2:** Comparision of means for TMT-A, TMT-B, d2-letter cancellation test, iCNV-amplitude and habituation of iCNV

**Sample (n = 33)**	**Mdiff**	**SDdiff**	**t**	**df**	**sig. (one-tailed)**
**TMT-A in seconds t1– t2**	2.51	8.06	1.79	32	.04*
**TMT-A in percentile rank t1– t2**	−10.00	27.39	−2.10	32	.02*
**TMT-B in seconds t1 – t2**	10.81	18.88	3.30	32	.00*
**TMT-B in percentile rank t1 – t2**	−18.94	26.69	−4.08	32	.00*
**Total number of finished items t1– t2**	−35.79	76.70	−2.68	32	.01*
**Total number of finished items less the number of mistakes t1– t2**	−21.09	43.33	−2.80	32	.01*
**Total number of mistakes t1 – t2**	-.64	10.46	-.35	32	.37
**iCNV**	-.75	2.11	−1.98	30	.03*
**Habituation**	-.38	.95	−2.21	30	.02*

n.s. = non-significant, M_Diff_ = difference in means; SD_Diff_ = difference in standard deviation; t = t-Wert; df = degrees of freedom; sig. = significance; *p < ,05;.

### Amplitude and habituation of the initial CNV

**Figure 3:**
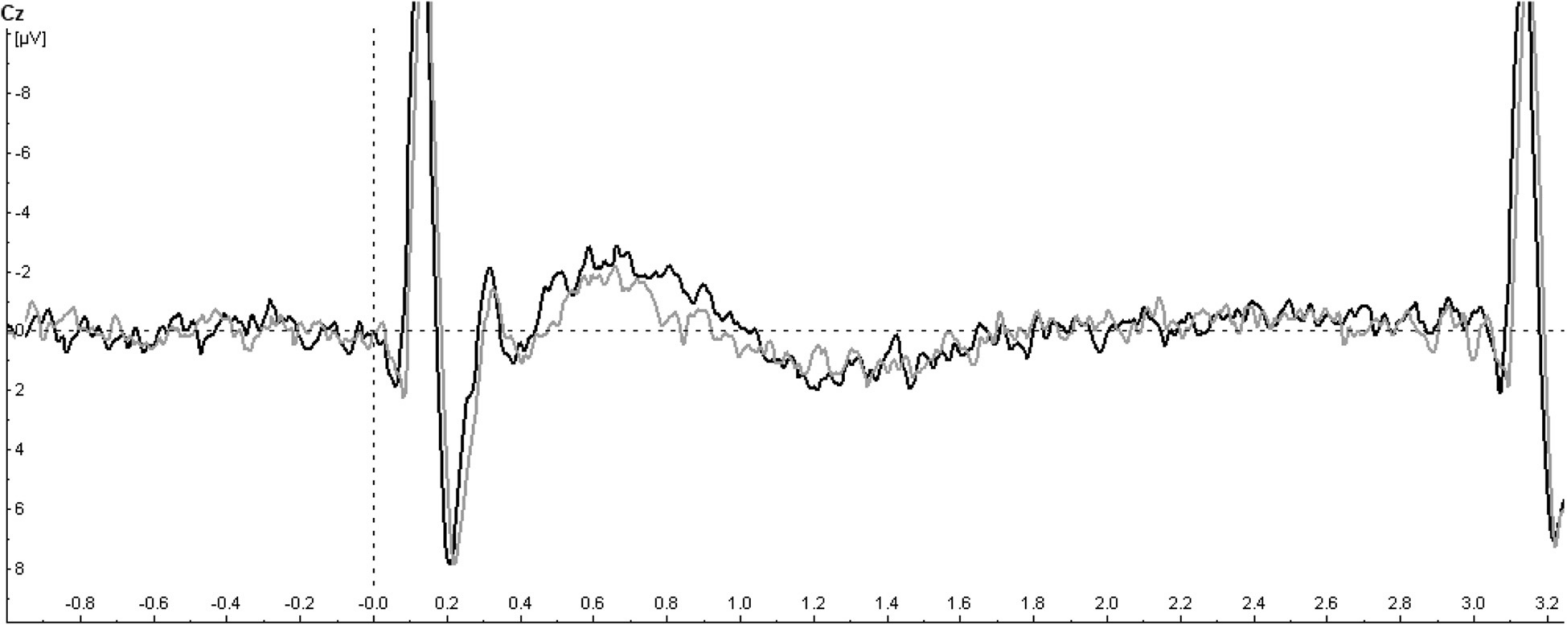
Grand average of CNV-amplitudes among all subjects before the training (black) and after the training (grey).

**Figure 4:**
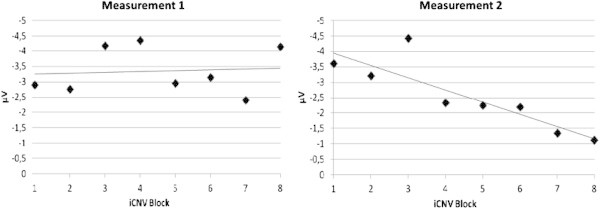
Differences of means for iCNV before the training (t1) and after the training (t2) for each patient.

**Figure 5:**
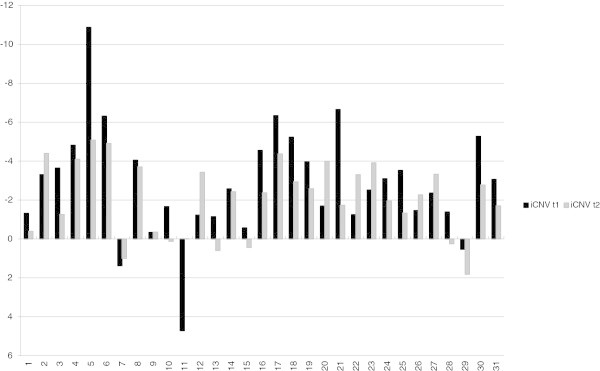
**Illustration of iCNV-dishabituation before the training (measurement 1) and iCNV- habituation after the training (measurement 2).** The figure shows mean amplitudes of the iCNV among the blocks of recording for all subjects and the regression line demonstrating the trend of dishabituation or habituation.

**Figure 6:**
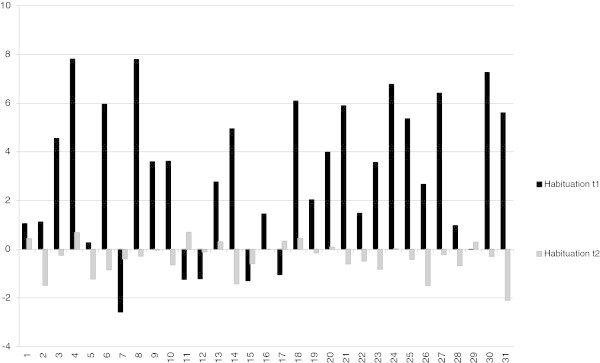
Differences of habituation coefficient before the training (t1) and after the training (t2) for each patient.

The effect of the training on the amplitude and habituation of the initial CNV is demonstrated in Table 2 and Figure 3 and iCNV is demonstrated in Figure 4 for each patient. Figure 5 represents the course of iCNV habituation over eight blocks of recording and the corresponding slope of linear regression. After the training, there was a significant reduction of the iCNV amplitude (t(30) = −1.98; p = .03 with M1 = −2.83 and M2 = − 2.08) and a significant increase of the iCNV habituation (t(30) = 2.21; p = .02 with, M1 = .02 and M2 = −.36) compared with the parameters obtained before the training. The reaction time showed a significant change (t(30) = −13,89; p = .00 with M1 414,25 and M2 428,14). Additionally, there was a significant correlation between the iCNV-amplitude and the physical working capacity (PWC 150 in km/h) as a parameter of physical fitness (in km/h: r = .60, p = .001; in watt: r = .56, p = .001). Figure 6 represents the course of iCNV habituation for each patient.

## Discussion and Conclusions

### Clinical and neurophysiological effects of the aerobic exercise training

The significant reduction of migraine attack frequency after aerobic exercise shows that a ten-week training program exerts an effect on the clinical course of migraine and may be used as an alternative treatment strategy. These results correspond with a great number of previous studies demonstrating a clear effect of sports and especially of aerobic exercises on migraine frequency and severity [14-28]. An increase of the fitness level might be one possible predictor for the reduction of migraine attack frequency [17]. Another predictor may be related to normalization of CNV parameters after sports. These findings are in line with previous neurophysiological research. It has been demonstrated repeatedly that migraine patients are characterized by increased amplitude and reduced habituation of the iCNV and that these abnormalities are most pronounced several days before a migraine attack representing attack anticipation and increased susceptibility to provoking agents [44-47]. Therefore, if any treatments would cause normalization of CNV parameters, these effects would reduce susceptibility to migraine attacks and frequency of attacks. Indeed, different pharmacological agents such as beta-blockers (propranolol and metoprolol), calcium antagonists (cyclandelate), and antiepileptic drugs (topiramate) and psychotherapy have cause a significant reduction of migraine frequency and contributed to normalization of the iCNV habituation [10-13]. Moreover, a specific neurofeedback training of self-regulation of slow cortical potentials (i.e. CNV) which was aimed to influence directly CNV habituation, led to a substantial clinical improvement of migraine and a clear normalization of iCNV habituation after the training [48]. The current study demonstrates that aerobic exercise training may influence clinical course of migraine through the same mechanisms as pharmacological and behavioral treatment options as well as neurofeedback, i.e. through normalization of cortical information processing in migraine.

There could be different explanations for that how aerobic exercise may affect the cortical information processing and, through it, the clinical course of migraine. It has been demonstrated that the aerobic exercise may increase or optimize level of several neurotransmitters such as serotonin, dopamine, acetylcholine and norepinephrine, activate the endocannabinoid system and endogenous opioidergic system, increase release of several neurotrophic factors related to better cognitive function, neurogenesis, angiogenesis and plasticity such as brain-derived neurotrophic factor (BDNF), the insulin-like growth factor (IGF-1), vascular endothelial growth factor (VEGF), calcitonine gene related peptide (CGRP), neurotrophin-3 and nerve growth factor (NGF), lower level of cortisol and optimize function of the hypothalamus-pituitary-adrenal (HPA) axis, as well as upregulate endothelial nitric oxide synthesis and improve angiogenesis and cerebral blood flow see for review [49,50]. Many of the mentioned neurotransmitters, neuropeptides and hormones play an important role in the pathogenesis of migraine [51]. We can only speculate about possible mechanisms of exercise in migraine. On the one hand, the exercise may reduce the pain perception through activation of opioidergic system [52]. This could have been the case, because the exercise in our study influenced the migraine duration more than the frequency of migraine. On the other hand, it could be suggested that the aerobic exercise caused an optimization of noradrenergic function, which resulted in an improved habituation, reduced susceptibility to migraine attacks and improved clinical course of migraine. This suggestion is based on previous findings demonstrating that the iCNV amplitude is closely related to the noradrenergic function [53,54] and the aerobic exercise may influence noradrenergic activity [55]. However, further studies are needed in order to proof hypotheses concerning clinical effects of sports on pathogenetic mechanisms of migraine.

In this study, migraine patients demonstrated a high information processing velocity (TMT-A at t1), split attention seems to be ordinary (TMT-B at t1). After aerobic endurance training, cognitive flexibility was improved. Patients featured an increased information processing velocity and an increased split attention as well as an increased selective attention ability measured by the d2-letter cancellation test. The processing time was increased but the number of taken mistakes didn’t changed significantly. These results indicate that participation in an endurance exercise programme improved the inadequate attention selection of the migraine patients. Stimuli could be filtered more adequate after training. To our knowledge there are no studies in the literature about migraine and exercise which tested attentional processes with instruments like TMT-A, TMT-B and d2 letter cancellation test. In regard to the current literature about exercise and cognitive processes [29-31,34], it can be concluded that physical exercise improves temporal processes, inhibitory control and response time. The interpretation of the results should be treated with caution because there was no proven correlation between the cardiopulmonary performance (PWC) and parameters of attention except for iCNV. It also has to be considered that the increased performance of TMT-A and TMT-B may be explained by a learning or repetition effects based on replication of the paper-pencil-test.

### Limitations of the study

The interpretability of results of this study is limited by several methodological aspects which have to be mentioned. Because different previous controlled studies have demonstrated a significant positive effect of sports and aerobic exercise training on headache characteristics [17 – 28], and the main focus of the current study was directed towards neurophysiological mechanisms underlying these positive clinical effects, we abstained from control conditions and chose an open study design. The missing control group remains an important limitation of the study. For this study, the appropriate control condition should include a group of patients who should be involved in a session sequence inducing placebo effect (the same number of sessions as in the aerobic exercise training, but with no nearly specified occupation during each session). Without an appropriate control condition, we cannot exclude that the demonstrated effects of the study may be associated with the placebo effect. We assume, however, that the placebo effect is less likely because in a previous similar study the exercise group showed a significant reduction of migraine attacks whereas the control group showed none [17]. Additionally, we can’t exclude a statistical regression to the mean (in this case the statistical phenomena that the subjects became less migraine attacks over time simply as a result of the variability in their sample) as a possible explanation for the reduction of migraine frequency. One other bias is related to the sample selection. This study was conducted with participants who were interested in exercise. However, the study with such a demanding exercise is only possible in motivated subjects. Note, even the subjects were interested in exercise, the study is characterized by a pretty high dropout rate of 46 percent. The main reasons (two out of three) for discontinuation were that the training three times weekly couldn’t be realized and/or the participants missed the training sessions more often than five times in total. Other causes were physical issues and missing measurements after the training. In one case migraine exacerbated and the training had to be interrupted.

Further limitations are related to the CNV methodology. CNV shows a periodicity depending on the migraine symptoms. The most pronounced amplitudes and the most expressed reduction of the iCNV habituation may be observed only several days before a migraine attack [44-46]. After an attack the CNV parameters normalize and do not differ from those in healthy subjects. If recordings will be carried out in different phases of the pain-free interval (for example, in the one group just before an attack and in another group after an attack), this may lead to a bias by comparing groups which will differ significantly. That’s why it is important to standardize CNV-recordings according to the time point of the pain-free interval. In this study it was intended to measure CNV-amplitudes four days after a migraine attack. In two cases this procedure couldn’t be realized because the persons suffered from very high-frequent migraine attacks. However, all EEGs were recorded during the interictal interval. Literature about CNV and migraine also pointed out that migraine attacks can be related to the feminine menstrual cycle [47]. In this study, the menstrual cycles as well as time from the recording to the next migraine attack were not registered. Therefore, the influence of the menstrual cycle as well as of periodic changes of neurophysiological parameters in migraine on results presented here cannot be excluded.

And finally, additional limitations are related to a narrow spectrum of clinical characteristics obtained in this study. As demonstrated previously, sports and aerobic exercise training may exert positive effects on psychological wellbeing, psychiatric co-morbidity, quality of life, and pain perception [17 – 28]. These secondary outcome measures might influence the clinical course of migraine and explain clinical effects of sports. However, these parameters were not collected in the current study, which focused more on neurophysiological mechanisms of aerobic exercise in migraine. Despite of limitation, this is one of the few clinical exploratory studies, which provides an evidence for influence of the aerobic exercise on cortical information processing in migraine and underlines the significance of sports in the clinical course of headaches.

### Clinical implications bullet points

In summary, the aim of this study was to investigate the influence of exercise on information processing and clinical course of migraine. To our knowledge it is the first study examining the influence of exercise on cortical information processing (iCNV-amplitude and habituation) in migraine. The results show an interaction between exercise, attention parameters and migraine. It can be suggested that our ten-week aerobic exercise training led to an improvement of attention, so that stimuli could be selected more adequate. Hence mmigraine as a disorder of central information processing reduced. This was demonstrated by decreased amplitudes and a normalized habituation of the iCNV in migraine.

## Competing interests

Parts of the study were supported by grant from the german migraine and headache society (Deutsche Migräne- und Kopfschmerzgesellschaft, DMKG). All authors declare that they have no competing interests. We thank Dr. med. A. Heinze (Schmerzklinik Kiel, Germany) for recruiting of migraine patients.

## Authors’ contributions

CHO, SD, MCE, WDG, MG, AK, UN, HS, MS and BW carried out the aerobic endurance study with patients with migraine, participated in the sequence alignment and drafted the manuscript. All authors read and approved the final manuscript.
